# Supplementary material for: The effects of exercise training on autonomic and hemodynamic responses to muscle metaboreflex in people living with HIV/AIDS: A randomized clinical trial protocol
Source: PLoS One. 2022 Mar 18;17(3):e0265516. doi: 10.1371/journal.pone.0265516 (PMC8932586; doi:10.1371/journal.pone.0265516)
Supplement: S1 File — (PDF) [file pone.0265516.s002.pdf]

University of Rio de Janeiro State

Institute of Physical Education and Sports

Research Project

HEMODYNAMIC AND AUTONOMIC RESPONSES TO MUSCLE ERGOREFLEX ACTIVATION IN PATIENTIS LIVING WITH HIV/AIDS: THE EFFECTS OF EXERCISE TRAINING

Research Team

Juliana Pereira Borges - Principal Investigator

Gabriel da Silva Gama - Associate Investigator

1 ABSTRACT

Introduction: People living with HIV have lower maximum cardiac output and attenuated pressure response to exercise compared to their healthy peers, which may be associated with damage to the neural mechanisms responsible for cardiovascular adjustment in these patients. Changes in muscle ergoreflex control can induce exercise intolerance and increased cardiovascular risk. Objective: To investigate the effect of physical training on autonomic and hemodynamic responses during muscle ergoreflex activation in patients living with HIV. Methods: Adults living with HIV without regular exercise of both sexes will be recruited, who will be randomized into a group submitted to physical training (HIV+/Active) and another control that will remain inactive (HIV+/Inactive). A reference group composed of non-HIV-infected and inactive (HIV-) subjects will be included. Active and Inactive HIV+ groups will be evaluated before and after the intervention, while HIV- only in the pre-intervention period. Physical training, carried out only by the HIV+/Active group, will consist of 12 weeks, with 3 weekly sessions of 60 minutes, aerobic and strength exercise with moderate intensity. In the evaluation, the subjects will be submitted to the mental stress Stroop test and the activation of the muscular ergoreflex through the protocol of circulatory restriction post-exercise (PECA) and passive exercise (PE). The PECA protocol will consist of 3 phases: 1) 2 minutes of isometric knee extension strength exercise at 30% of maximum voluntary contraction; 2) 2 minutes of circulatory restriction of the exercised limb, performed through a pressure cuff inflated to 240 mmHg; and 3) 3 minutes of recovery without circulatory restriction. On the same day, after 30 minutes and in a counterbalanced way, the PE protocol will be performed, which will consist of: 1) 2 minutes of knee extension exercise performed without load and passively; 2) 5 minutes of post-exercise recovery without circulatory restriction. During the mental stress test and PECA and PE protocols, autonomic and hemodynamic responses will be evaluated through heart rate variability and photoplethysmography; respectively.

Keywords: AIDS, physical training, ergoreflex, metaboreflex, pressure reflex during exercise.

2 Aims

2.1 Major Aim

To investigate the impact of physical training on hemodynamic and autonomic responses during ergoreflex muscle activation in patients with HIV.

2.2 Specific aims

1) Compare hemodynamic and autonomic responses between men and women with HIV vs. healthy controls during muscle ergoreflex activation;
2) Compare hemodynamic and autonomic responses between men and women with HIV vs. healthy controls during mental stress testing;
3) To investigate the impact of physical training on hemodynamic and autonomic responses in HIV patients during rest and mental stress testing;
4) To investigate the blood lactate concentration before and after physical training in patients with HIV;
5) To investigate baroreflex sensitivity before and after physical training in HIV patients.

3 METHODS

3.1 Participants

The study sample will be composed of adults living with and without HIV of both sexes. The group of people living with HIV will be recruited through social media and visits to the Infectious Parasitic Diseases Outpatient Clinic of Hospital Pedro Ernesto, State University of Rio de Janeiro (HUPE/UERJ). Subjects without HIV will be recruited through social media and outreach within the UERJ staff. All patients must be aged between 30 and 60 years, at least 5 years of HIV infection, use cART for at least 3 years, be asymptomatic, and free from opportunistic diseases during the study period. Subjects who practice regular exercise (3 or more weekly sessions of exercise for at least 30 min), with evidence of coronary artery disease, ischemic disease, pulmonary disease, diabetes mellitus, Chagas disease, tuberculosis, malnutrition, dehydration, heart failure, hypertension, patients with pacemakers and taking antidepressant, antiarrhythmic or

antihypertensive medication, especially beta-blockers will be excluded from the study. All patients will be volunteers and will sign an informed consent form (Appendix 1).

3.2 Experimental Design

The study will consist of a controlled and randomized clinical trial. Patients living with HIV included in the study will be randomized by a “blind” collaborator using a random code generator (www.randomization.com) into a group undergoing 12 weeks of physical training as described below and another control group that will remain inactive for the duration the study period. A reference group composed of healthy subjects matched for age and sex with HIV groups will be included and evaluated only in the pre-intervention period. Before and after physical training, the groups with HIV will undergo evaluations, which will be conducted in two visits to the Physical Activity and Health Promotion Laboratory (LABSAU) of UERJ, carried out on two non-consecutive days, in the morning between to 9 and 11 h in a room with controlled temperature between 22°-24°C and relative humidity between 60-70%.

On the first visit, the subjects will read the informed consent form and, if they agree to participate, they will be evaluated for habitual physical activity, body mass and height, using a mechanical scale with a precision of 100g (Cambé, Rolândia, Brazil) and stadiometer (Sanny, São Paulo, Brazil); respectively. After resting in the supine position for 30 min, a mental stress test (Stroop test) will be applied to assess hemodynamic and autonomic parameters for the activation of the central nervous system independent of the ergoreflex (efferent response). At the end of the session, the maximum force of unilateral knee extension will be evaluated through up to 5 attempts (with an interval of 2 minutes between them), using an leg extension machine (Techynogym Selection, Cesena, ITA).

In the second visit, the evaluation of hemodynamic parameters and autonomic modulation during ergoreflex activation will be performed through the methods of post-exercise circulatory arrest (PECA) and passive exercise (PE), described below. For both visits, volunteers will be recommended not to consume food in the two hours prior to the tests, not to drink coffee in the 12 hours before, not to engage in physical activity and/or to consume alcoholic beverages in the 48 hours preceding the assessment protocol. Hemodynamic parameters and autonomic modulation will be evaluated through photoplethysmography and heart rate variability; respectively. Figure 1 illustrates the experimental design of the study.

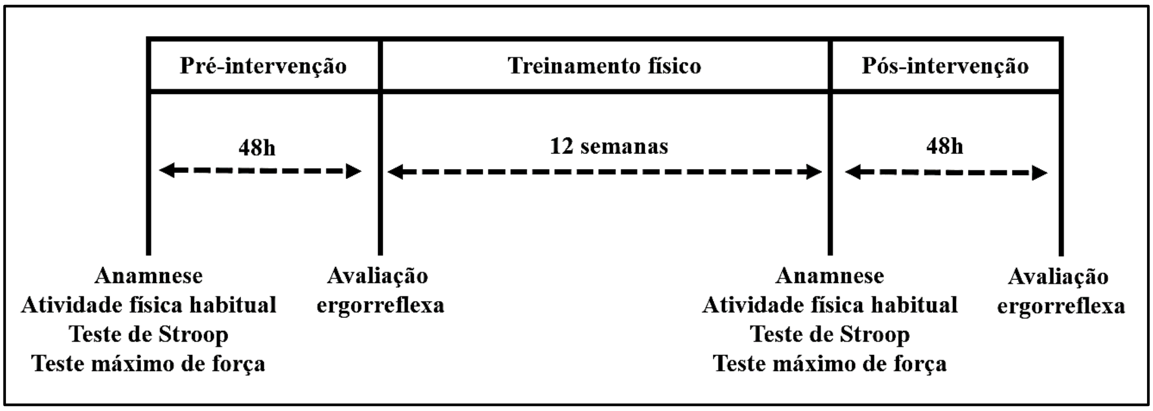

Fig. 1: Experimental design.

3.3 Physical Training

The physical training will last 12 weeks and will be held at the Training Center of the Physical Activity and Health Promotion Laboratory (LABSAU) at UERJ. The training will consist of aerobic and resistance exercise, performed for 3 weekly sessions, lasting 60 min per session. Aerobic exercise will be performed on a treadmill or ergometric bicycle, with an intensity range corresponding to 60-70% of the reserve heart rate (HR), determined by the Karvonen equation: reserve HR = [(maximum HR – HR rest) x 60 at 70%] + HR rest. Resistance training will consist of 8 to 10 exercises performed in 3 sets of 10 to 12 repetitions maximum for the main muscle groups (quadriceps, hamstrings, gastrocnemius/soleus, pectorals, back, trapezius, shoulders, triceps, biceps and abdominals).

3.4 Assessment of habitual physical activity

Habitual physical activity will be assessed using the Baecke questionnaire, previously validated for people living with HIV [1]. The Baecke is an instrument that assesses habitual physical activity in the last 12 months, through 8 questions that address on a quali-quantitative scale the magnitude of occupational physical activities, physical exercises during leisure time and physical activities performed in leisure and locomotion.

3.5 Stroop test

To assess the integrity of the efferent response of the autonomic system, sympathetic stimulation independent of the ergoreflex will be performed using the Stroop mental stress test [2]. This test consists of 3 phases: 1) presentation of a word poster; 2) presentation of a poster of words with corresponding colors; 3) presentation of a poster

of words with non-corresponding colors, as shown in Figure 2. In the first phase, the subject must read the words aloud, and in the second and third phase, the subject must speak the colors aloud in that the words shown on the poster are printed. All phases will consist of a total of 25 words.

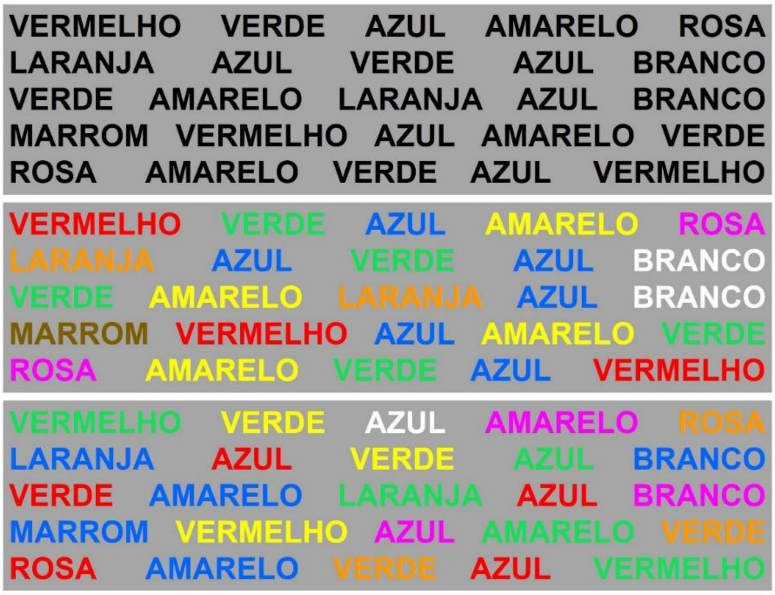

Fig. 2: Poster used during Stroop test. The top poster is used in the first phase, the middle one in the second and the bottom one in the third phase.

3.6 Activation of ergoreflex activity

To assess ergoreflex activity, a digital sphygmomanometer (Omron HEM - 7113, Kyoto, JPN) will be positioned around the proximal third of the subject's non-dominant arm, where blood pressure measurements will be taken every minute of the protocol. To perform vascular occlusion, a digital sphygmomanometer (Hokanson, TD312 Calculating Cuff Inflator, USA) will be positioned around the proximal third of the thigh of the dominant limb.

After instrumentation, the ergoreflex activity will be evaluated through two protocols, PECA and PE that will analyze the behavior of the muscle metabo- and mechanoreflex; respectively. Both protocols will have a total duration of 11 minutes each, and will be performed in random and counterbalanced order, with a 30-min interval between them. As shown in Figure 3, in PECA, the individuals will initially remain at rest for 3 minutes before performing an isometric exercise of unilateral knee extension of the dominant limb with 30% of the maximum force for 3 min, followed by a period of 2 minutes of ischemia of the exercised limb, performed through a cuff inflated to 240 mmHg, followed by another 3 minutes of recovery without circulatory restriction, totaling

5 minutes of post-exercise recovery. The PE protocol will start with 3 minutes of rest prior to performing the unilateral knee extension of the dominant limb performed passively and rhythmically at 1 movement every 2 seconds for 3 minutes, totaling 90 movements. Then, the volunteer will go through a period of 5 minutes of post-exercise recovery without circulatory restriction, ending the protocol. During each PECA and PE protocol, autonomic and hemodynamic responses will be assessed every minute.

During both protocols, volunteers will be instructed to avoid the Valsalva maneuver, and not to perform any movement other than knee extension.

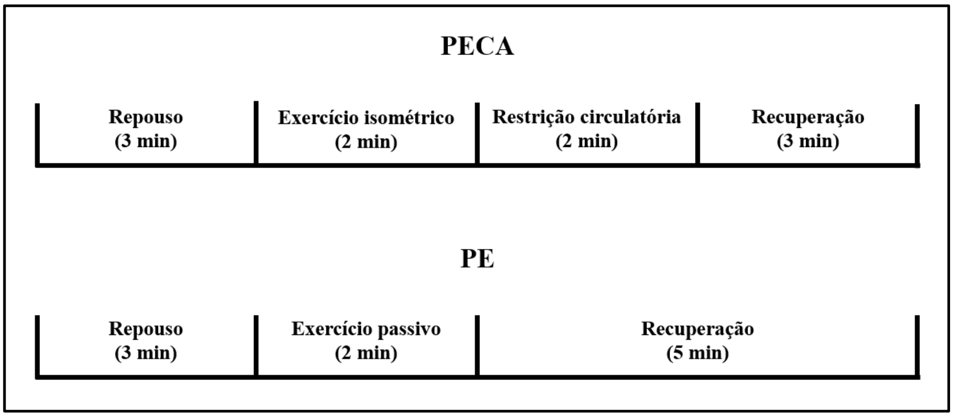

Fig. 3: Ergoreflex assessment protocols. PECA, postexercise circulatory arrest; PE, passive exercise.

The metabolic stress will be verified through the quantification of blood lactate during the 3rd min of rest, 2nd and 5th min of post-exercise recovery in PECA and PE using a YSI 2700 analyzer (Yellow Springs©, OH, USA).

3.7 Hemodynamic and autonomic modulation assessment

Hemodynamic parameters and autonomic modulation will be continuously evaluated during the 30 min of rest, Stroop test and activation of the ergoreflex. Hemodynamic responses will be continuously assessed using photoplethysmography (Finometer PRO, Finapress Medical Systems, Enschede, NL), which allows for the non-invasive, beat-to-beat assessment of heart rate, systolic blood pressure, diastolic blood pressure, pressure mean arterial, stroke volume, cardiac output, peripheral vascular resistance and baroreflex sensitivity.

Autonomic modulation will be evaluated through heart rate variability (HRV), obtained by analyzing the RR intervals recorded by a heart rate monitor (Polar RS 800

XC, Kempele, FIN). The analysis of the data obtained for the HRV will be performed in the domains of time and frequency, through the Kubios HRV program (Version 2.2, Kuopio, Finland). To measure the HRV in the frequency domain, the spectral potential will be estimated by the fast Fourier transform algorithm, considering for analysis purposes the high (HF: 0.15 to 0.40Hz), low frequency (LF: 0) components .04 to 0.15Hz) and the ratio between the components (LF/HF). The values of the different components will be calculated in the form of normalized units (n.u.), dividing the power of each component by the total power, subtracted from the value of the component itself and multiplied by 100.

3.8 Statistical analysis

The sample size was calculated by the GPower software (version 3.0.10, University of Kiel, Kiel, Germany) based on a difference of 6.7 mmHg for the metaboreflex gain (standard deviation of 10 mm Hg). Assuming 80% potency, 5% significance level and increasing sample size by 50% due to follow-up losses over the 12 weeks of the study, 22 patients in each group were determined as needed.

To evaluate the ergoreflex activity, the average of the last two minutes of recovery obtained in the PECA and PE protocols will be considered. Then, the difference between PECA and PE will be calculated. This procedure will allow the assessment of metaboreflex gain, that is, the response due to metaboreflex activity. Differences between groups will be assessed by three-way factorial ANOVA (group vs time vs training) followed by Tukey's post hoc test, when appropriate. Statistical analysis will be performed using commercially available software (Stata 13.0), assuming a significant level established at $P < 0.05$ in all cases.

4 SCHEDULE

The activities that will be carried out in each of the semesters during the execution of the project are presented below.

Table 1. Schedule of execution

**Activities/Quarters**	**1st year**		**2nd year**		**3rd year**		**4th year**	
Bibliographic Update								
Obtaining approval from the ethics committee								
Pilot Testing								
Intervention (physical training)								
Data collection and analysis								
Disclosure of preliminary results / Qualification								
Original article submission								
Thesis writing and presentation								

5 REFERENCES

1. Florindo, A.A., et al., *Validity and reliability of the Baecke questionnaire for the evaluation of habitual physical activity among people living with HIV/AIDS.* Cadernos de saude publica, 2006. **22**: p. 535-541.
2. Stroop, J.R., *Studies of interference in serial verbal reactions.* Journal of experimental psychology, 1935. **18**(6): p. 643.

6 APPENDIX 1 – INFORMED CONSENT FORM

PATIENT CODE _____________

INFORMATION FORM TO PATIENTS

"Document prepared in application of the recommendations of the Declaration of Helsinki"

You are being invited to participate in a research study entitled "HEMODYNAMIC AND AUTONOMIC RESPONSES TO MUSCLE ERGORREFLEX ACTIVATION IN PATIENTS LIVING WITH HIV: EFFECTS OF EXERCISE TRAINING" organized by the Physical Activity and Health Promotion Laboratory at the Institute of Physical Education and Sports at the State University of Rio de Janeiro (UERJ) under the responsibility of researchers Juliana Borges and Gabriel Gama.

1. STUDY AIMS

Major aim

To investigate the autonomic and hemodynamic response during metaboreflex activation and the impact of physical training on this response in people living with HIV.

Specific aims

• Verify the autonomic and hemodynamic responses during mechanoreflex activation and the impact of physical training on this response in people living with HIV;
• Verify the baroreflex function during ergoreflex activation and the impact of training on this response in people living with HIV;
• Investigate the impact of exercise training on autonomic modulation in people living with HIV;
• Compare the hemodynamic and autonomic responses between men and women with HIV to activation of the muscle metaboreflex;
• Correlate the hemodynamic and autonomic responses to the activation of the muscle metaboreflex with markers of the patients' physical fitness.

2. PROCEDURES

You will be assisted at the Physical Activity and Health Promotion Laboratory of the Physical Education and Sports Institute of UERJ (Address: João Lira Filho Pavilion, 8th floor. Rua São Francisco Xavier 524, Maracanã) on two non-consecutive days by physical education professionals, which will explain the objectives of this proposed study and signature of the Free and Informed Consent Term (TCLE).

The following procedures will be performed:

Evaluations:

- Anamnesis with weight and height measurements;

- Application of the Baecke questionnaire to determine the level of habitual physical activity;
- Verification of autonomic modulation at rest, and during the application of the Stroop color and word test through heart rate variability;
- Performing the maximum strength test for unilateral knee extension.
- Application of the ergoreflex assessment protocol, which consists of performing the unilateral knee extension exercise (leg extension machine) in an isometric and passive way, with and without circulatory restriction through a pressure cuff positioned in the proximal third of the subject's leg;
- Verification of autonomic, hemodynamic, and metabolic responses to the ergoreflex assessment protocol, through heart rate variability, photoplethysmography (similar to electrocardiogram) and blood lactate collection, respectively.

<u>Exercise Training:</u>

After the second visit for evaluations, we will start the physical training program that will consist of aerobic, resistance and flexibility exercises with a total duration of 12 weeks, being performed 3 times a week.

3. BENEFITS AND POTENTIAL RISKS

Some adverse effects, although uncommon, are described during some exams and tests:

- Maximal muscle strength testing can, in some people, cause delayed muscle soreness;
- Circulatory restriction can, in some people, cause momentary discomfort (such as cramp). The presence of any discomfort will cause the test to stop immediately.
- Blood lactate collection can, in some people, because it is a minimally invasive procedure, cause a slight momentary discomfort in the volunteer's finger.

As a benefit, a complete control analysis of blood pressure, heart rate, among other hemodynamic variables will be performed, free of charge for the volunteer.

4. VOLUNTARY DISCONTINUATION OF THE STUDY

You can refuse to participate in the research, and even if you decide to participate, you can withdraw from the study at any time without needing to provide an explanation. This will not affect the treatment or the doctor-patient relationship with your doctor.

5. PATIENT PROTECTION AND ETHICS

This study will be carried out in accordance with the principles established in the Declaration of Helsinki and subsequent amendments (2000) and in accordance with Good Medical Practice (ICH/E6).

6. CONFIDENTIALY

Study confidentiality is guaranteed by the fact that your patient code (a number and initials) will appear in all written study documents. The information collected will be processed by computer and can be checked by health authorities.

If you have any questions about this study, please contact the researcher responsible for the study, Profa. Dr. Juliana Pereira Borges (LABSAU), on the telephone (21) 2334-0775.

Researcher responsible for the Research Project
Prof. Dr. Juliana Borges (LABSAU)

Research Project Execution
Prof. Me. Gabriel da Silva Gama (LABSAU)

INVESTIGATOR SESSION (to be completed by the investigator)

Name:...

Contact Person: Juliana Pereira Borges Phone Number: (021) 2334-0775
I confirm that I have explained the experiment in detail to the patient. I informed him of the information form and answered all his questions related to the study.

Ass.:……………………………………………………………….. Date: ....

VOLUNTEER SESSION (to be completed by the volunteer)

Name:……………………………………………………………………………......................

Address:...

I received, read and understood the information form for the study designated above. I was also given adequate explanations about the clinical trial, its purposes, risks, my rights as a patient, and what I will have to do and submit to. I was given every opportunity to ask anything before making any decision. I may need additional information at any time from the investigator. I know that my decision to participate in this study is entirely up to me, and that I have the right to change my mind at any time during the course of the study without this affecting my treatment in the future. I understand that the experiment or my participation in it can be interrupted at any time by the investigator. I also understand that access to relevant information about my patient data may be required as part of the study and that data collected during the study may be checked by health authorities and sponsor representatives in accordance with current legislation.

I understand that my anonymity will be preserved in the strictest confidence. The data recorded during the study will be processed by computer and I understand that I will have the right, if I wish, to access the computerized data. I have received a copy of this document and have been informed that a copy will be kept confidentially by the study sponsor. Therefore, I give my consent to be part of this study.

Assinature:...

Dated by volunteer:………/…………./…..…….

University of Rio de Janeiro State
Institute of Physical Education and Sports

Amendment to the Original Research Project

HEMODYNAMIC AND AUTONOMIC RESPONSES TO MUSCLE ERGOREFLEX ACTIVATION IN PATIENTIS LIVING WITH HIV/AIDS: THE EFFECTS OF EXERCISE TRAINING

Research Team

Juliana Pereira Borges - Principal Investigator

Gabriel da Silva Gama - Associate Investigator

The present request for amendment is necessary due to 3 changes in the study protocol found to be necessary based on the pilot tests: 1) the passive exercise (PE) protocol for mechanoreflex assessment was withdrawn, and for this reason, only one visit to the laboratory will be carried out, instead of two visits as originally presented; 2) the knee extension exercise was replaced by the handgrip exercise; and 3) the evaluation of the muscle metaboreflex will be carried out without and with the application of a capsaicin-based balm, which is commercially sold for the treatment of osteoarthritis, in order to amplify the activation of the muscle metaboreflex prior to PECA.
