# Supplementary material for: The effects of exercise training on autonomic and hemodynamic responses to muscle metaboreflex in people living with HIV/AIDS: A randomized clinical trial protocol
Source: PLoS One. 2022 Mar 18;17(3):e0265516. doi: 10.1371/journal.pone.0265516 (PMC8932586; doi:10.1371/journal.pone.0265516)
Supplement: S2 File — (PDF) [file pone.0265516.s003.pdf]

Universidade do Estado do Rio de Janeiro
Instituto de Educação Física e Desportos

PROJETO DE PESQUISA

RESPOSTAS HEMODINÂMICAS E AUTONÔMICAS À ATIVAÇÃO ERGORREFLEXA MUSCULAR EM PACIENTES VIVENDO COM HIV/AIDS: EFEITOS DO TREINAMENTO FÍSICO

Equipe de Pesquisa

Juliana Pereira Borges - Investigador Principal

Gabriel da Silva Gama - Investigador Associado

1 RESUMO

Introdução: Pessoas vivendo com HIV apresentam menor débito cardíaco máximo e resposta pressórica atenuada ao exercício em relação aos seus pares saudáveis, que pode estar associado a danos nos mecanismos neurais responsáveis pelo ajuste cardiovascular nesses pacientes. Alterações no controle ergorreflexo muscular podem induzir intolerância ao exercício e maior risco cardiovascular. **Objetivo:** Investigar o efeito do treinamento físico sobre respostas autonômicas e hemodinâmicas durante ativação do ergorreflexo muscular em pacientes vivendo com HIV. **Métodos:** Serão recrutados adultos vivendo com HIV sem exercício físico regular de ambos os sexos, que serão randomizados em um grupo submetido a treinamento físico (HIV+/Ativo) e outro controle que permanecerá inativo (HIV+/Inativo). Um grupo de referência composto de sujeitos não-infectados pelo HIV e inativos (HIV-) será incluído. Os grupos HIV+ Ativo e Inativo serão avaliados antes e após a intervenção, enquanto o HIV- somente no período pré-intervenção. O treinamento físico, realizado somente pelo grupo HIV+/Ativo, será composto por 12 semanas, com 3 sessões semanais de 60 minutos, de exercício aeróbio e de força com intensidade moderada. Na avaliação, os sujeitos serão submetidos ao teste de Stroop de estresse mental e à ativação do ergorreflexo muscular através do protocolo de restrição circulatória pós-exercício (PECA) e exercício passivo (PE). O protocolo de PECA será composto por 3 fases: 1) 2 minutos de exercício de força isométrica de extensão de joelho a 30% da contração voluntária máxima; 2) 2 minutos de restrição circulatória do membro exercitado, realizada através de um manguito de pressão inflado a 240 mmHg; e 3) 3 minutos de recuperação sem a restrição circulatória. No mesmo dia, após 30 minutos e de forma contrabalanceada, será realizado o protocolo PE, que será composto por: 1) 2 minutos de exercício de extensão de joelho realizado sem carga e de maneira passiva; 2) 5 minutos de recuperação pós-exercício sem restrição circulatória. Durante o teste de estresse mental e protocolos PECA e PE, as respostas autonômicas e hemodinâmicas serão avaliadas através da variabilidade da frequência cardíaca e fotoplestimografía; respectivamente.

Palavras-Chave: AIDS, treinamento físico, ergorreflexo, metaborreflexo, reflexo pressórico ao exercício.

2 OBJETIVOS

2.1 Objetivo Geral

Investigar o impacto do treinamento físico sobre respostas hemodinâmicas e autonômicas durante ativação ergorreflexa muscular em pacientes com HIV.

2.2 Objetivos específicos

1) Comparar as respostas hemodinâmicas e autonômicas entre homens e mulheres com HIV vs. controles saudáveis durante ativação do ergorreflexo muscular;
2) Comparar respostas hemodinâmicas e autonômicas entre homens e mulheres com HIV vs. controles saudáveis durante teste de estresse mental;
3) Investigar o impacto do treinamento físico sobre respostas hemodinâmicas e autonômicas em pacientes com HIV durante o repouso e teste de estresse mental;;
4) Investigar a concentração de lactato sanguíneo antes e após treinamento físico em pacientes com HIV;
5) Investigar a sensibilidade barorreflexa antes e após treinamento físico em pacientes com HIV.

3 MÉTODOS

3.1 Amostragem

A amostra do estudo será composta por adultos vivendo com e sem HIV de ambos os sexos. O grupo de pessoas vivendo com HIV será recrutado através de mídias sociais e visitas ao Ambulatório de Doenças Infecciosas Parasitárias do Hospital Pedro Ernesto da Universidade do Estado do Rio de Janeiro (HUPE/UERJ). Os sujeitos sem HIV serão recrutados através de mídia social e divulgação dentro do quadro de funcionários da UERJ. Todos os pacientes deverão possuir idade entre 30 e 60 anos, ao menos 5 anos de infecção por HIV, fazer uso de cART por pelo menos 3 anos, ser assintomáticos, e livres de doenças oportunistas no período do estudo. Serão excluídos do estudo sujeitos que pratiquem exercício regular (3 ou mais sessões semanais de exercício por pelo menos 30 min), com evidências de doença arterial coronariana, doença isquêmica, doença pulmonar, diabetes mellitus, doença de chagas, tuberculose, desnutrição, desidratação, insuficiência cardíaca, hipertensão, portadores de marcapasso e fazendo uso de

medicação antidepressiva, antiarrítmica ou anti-hipertensiva, especialmente betabloqueadores. Todos os pacientes serão voluntários e assinarão um termo de consentimento livre e esclarecido (ANEXO 1).

3.2 Desenho Experimental

O estudo terá caráter de ensaio clínico controlado e randomizado. Os pacientes vivendo com HIV incluídos no estudo serão randomizados por um colaborador "cego" usando um gerador de códigos aleatórios (www.randomization.com) em um grupo submetido a 12 semanas de treinamento físico conforme descrito abaixo e outro grupo controle que permanecerá inativo durante o período do estudo. Um grupo de referência composto de sujeitos saudáveis pareados por idade e sexo aos grupos com HIV será incluído e avaliado somente no período pré-intervenção. Antes e após o treinamento físico, os grupos com HIV serão submetidos às avaliações, que serão conduzidas em duas visitas ao Laboratório de Atividade Física e Promoção à Saúde (LABSAU) da UERJ, realizadas em dois dias não consecutivos, no período da manhã entre às 9 e 11 h em uma sala com temperatura controlada entre 22°-24°C e umidade relativa do ar entre 60-70%.

Na primeira visita, os sujeitos farão a leitura do termo de consentimento livre e esclarecido e caso concordem em participar, serão avaliados quanto a atividade física habitual, massa corporal e estatura, através de balança mecânica com precisão de 100g (Cambé, Rolândia, Brasil) e estadiômetro (Sanny, São Paulo, Brasil); respectivamente. Após repouso em decúbito dorsal durante 30 min, será aplicado um teste de estresse mental (teste de Stroop) para avaliação de parâmetros hemodinâmicos e autonômicos à ativação do sistema nervoso central independente do ergorreflexo (resposta eferente). Ao fim da sessão, será avaliada a força máxima de extensão de joelho unilateral por meio de até 5 tentativas (com intervalo de 2 minutos entre elas), utilizando uma cadeira extensora (Techynogym Selection, Cesena, ITA).

Na segunda visita, será realizada a avaliação dos parâmetros hemodinâmicos e modulação autonômica durante ativação ergorreflexa através dos métodos de restrição circulatória pós-exercício (em inglês: *postexercise circulatory arrest*, PECA) e exercício passivo (em inglês: *passive exercise*, PE), descritas abaixo. Para ambas as visitas, os voluntários serão recomendados a não consumir alimentos nas duas horas anteriores aos testes, não ingerirem café nas 12 horas anteriores, não realizarem atividade física e/ou consumam bebida alcoólica nas 48 horas precedentes ao protocolo de avaliação.

Os parâmetros hemodinâmicos e modulação autonômica serão avaliados por meio de fotopletismografia e variabilidade da frequência cardíaca; respectivamente. A Figura 1 ilustra o desenho experimental do estudo.

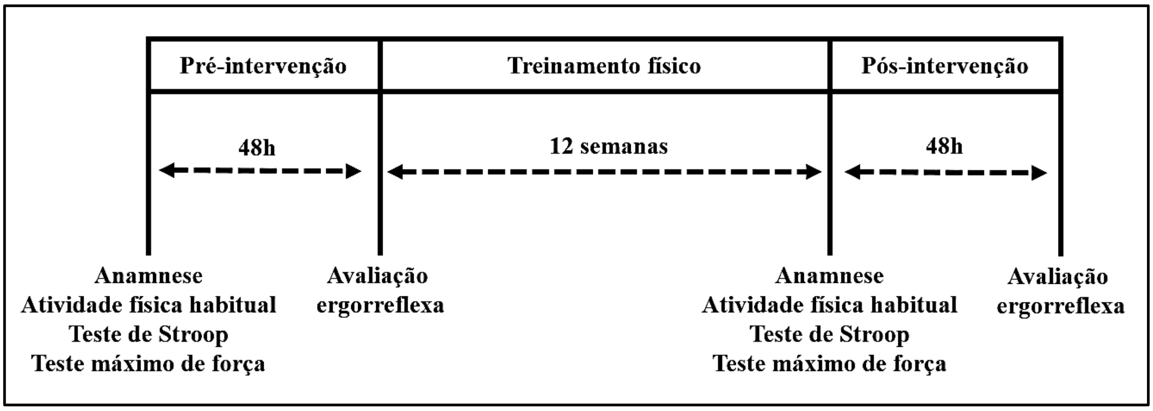

Fig. 1: Desenho experimental.

3.3 Treinamento Físico

O treinamento físico terá duração de 12 semanas e será realizado no Centro de Treinamento do Laboratório de Atividade Física e Promoção da Saúde (LABSAU) da UERJ. O treinamento consistirá em exercício aeróbio e resistido, realizado por 3 sessões semanais, com duração de 60 min por sessão. O exercício aeróbio será realizado em esteira ou bicicleta ergométrica, com faixa de intensidade correspondente a 60-70% da frequência cardíaca (FC) de reserva, determinada pela equação de Karvonen: FC de reserva = [(FC máxima – FC repouso) x 60 a 70%] + FC repouso. O treinamento resistido consistirá de 8 a 10 exercícios realizados por 3 séries de 10 a 12 repetições máximas para os principais grupamentos musculares (quadríceps, posteriores de coxa, gastrocnêmios/ sóleos, peitorais, costas, trapézio, ombros, tríceps, bíceps e abdominais).

3.4 Avaliação da atividade física habitual

A atividade física habitual será avaliada por meio do questionário Baecke, já validado anteriormente em pessoas vivendo com HIV [1]. O Baecke se trata de um instrumento que avalia a atividade física habitual nos últimos 12 meses, através de 8 questões que abordam em escala quali-quantitativa a magnitude de atividades físicas ocupacionais, exercícios físicos durante o tempo de lazer e atividades físicas realizadas no lazer e locomoção.

3.5 Teste de Stroop

Para avaliação da integridade da resposta eferente do sistema autonômico será realizada estimulação simpática independente do ergorreflexo através do teste de estresse mental de Stroop [2]. Esse teste consiste em 3 fases: 1) apresentação de um cartaz de palavras; 2) apresentação de um cartaz de palavras com cores correspondentes; 3) apresentação de um cartaz de palavras com cores não correspondentes, conforme demonstrado na Figura 2. Na primeira fase, o avaliado deverá ler as palavras em voz alta, e na segunda e terceira fase, o avaliado deverá falar em voz alta as cores em que estão impressas as palavras apresentadas no cartaz. Todas as fases serão compostas por um total de 25 palavras.

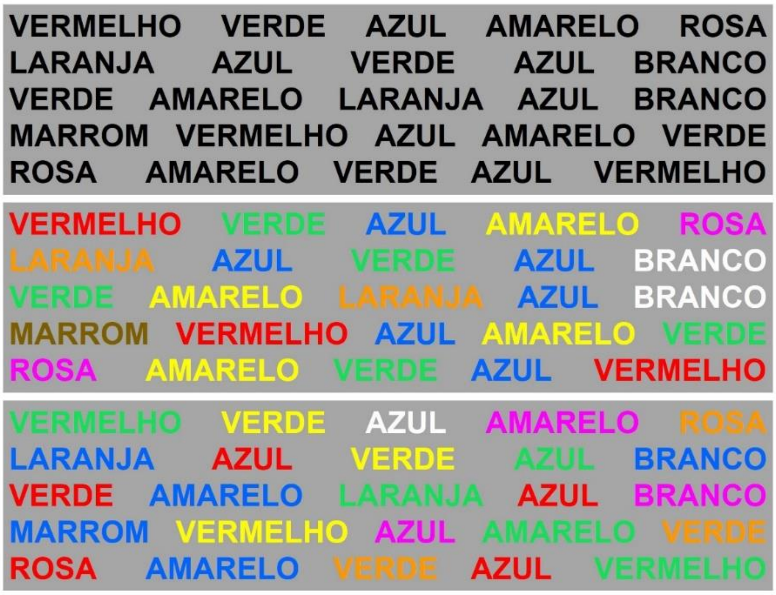

Fig. 2: Cartaz utilizado durante teste de Stroop. O cartaz superior é utilizado na primeira fase, o do meio na segunda e o inferior na terceira fase.

3.6 Ativação da atividade ergorreflexa

Para a avaliação da atividade ergorreflexa, um esfigmomanômetro digital (Omron HEM – 7113, Kyoto, JPN) será posicionado entorno do terço proximal do braço não-dominante do sujeito onde serão realizadas as medidas de pressão arterial a cada minuto do protocolo. Para a realização da oclusão vascular, um esfigmomanômetro digital (Hokanson, TD312 Calculating Cuff Inflator, EUA) será posicionado entorno do terço proximal da coxa do membro dominante.

Após a instrumentação, a atividade ergorreflexa será avaliada através de dois protocolos, PECA e PE que analisarão o comportamento do metabo- e mecanorreflexo muscular; respectivamente. Ambos os protocolos terão duração total de 11 minutos cada,

e serão realizados em ordem randômica e contrabalanceada, com 30 min de intervalo entre eles. Conforme demonstrado na Figura 3, em PECA, inicialmente os indivíduos permanecerão em repouso por 3 minutos antes de realizarem um exercício isométrico de extensão unilateral de joelho do membro dominante com 30% da força máxima durante 3 min, seguido por um período de 2 minutos de isquemia do membro exercitado, realizada através de um manguito inflado a 240 mmHg, seguido por mais 3 minutos de recuperação sem restrição circulatória, totalizando 5 minutos de recuperação pós-exercício. O protocolo PE será iniciado com 3 minutos de repouso prévio à realização da extensão unilateral de joelho do membro dominante realizada de forma passiva e ritmada a 1 movimento a cada 2 segundos durante 3 minutos, totalizando 90 movimentos. Em seguida, o voluntário passará por um período de 5 minutos de recuperação pós-exercício sem restrição circulatória, finalizando o protocolo. Durante cada protocolo PECA e PE, as respostas autonômicas e hemodinâmicas serão avaliadas a cada minuto.

Durante ambos os protocolos, os voluntários serão orientados a evitar a manobra de Valsalva, e não realizar qualquer movimento a não ser a extensão de joelho.

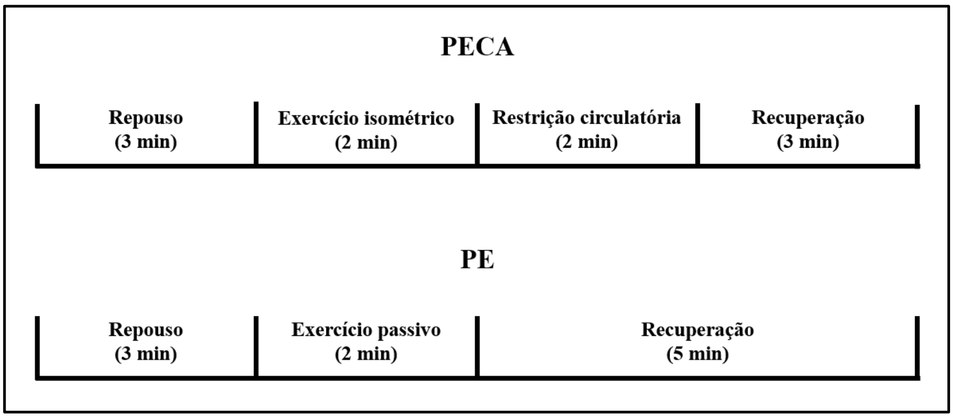

Fig. 3: Protocolos de avaliação ergorreflexa. PECA, postexercise circulatory arrest; PE, passive exercise.

O estresse metabólico será verificado por meio da quantificação do lactato sanguíneo durante o 3º min do repouso, 2º e 5º min da recuperação pós-exercício em PECA e PE através de analisador YSI 2700 (Yellow Springs©, OH, USA).

3.7 Avaliação hemodinâmica e da modulação autonômica

Os parâmetros hemodinâmicos e modulação autonômica serão avaliados continuamente durante os 30 min de repouso, teste de Stroop e ativação do ergorreflexo.

As respostas hemodinâmicas serão avaliadas de forma contínua através de fotoplestimografía (Finometer PRO, Finapress Medical Systems, Enschede, NL), que permite a avaliação de forma não invasiva batimento-a-batimento da frequência cardíaca, pressão arterial sistólica, pressão arterial diastólica, pressão arterial média, volume sistólico, débito cardíaco, resistência vascular periférica e sensibilidade barorreflexa.

A modulação autonômica será avaliada através da variabilidade da frequência cardíaca (VFC), obtida pela análise dos intervalos RR registrados por cardiofrequêncímetro (Polar RS 800 XC, Kempele, FIN). A análise dos dados obtidos para a VFC será realizada nos domínios do tempo e da frequência, através do programa Kubios HRV (Versão 2.2, Kuopio, Finlândia). Para aferição da VFC no domínio da frequência, o potencial espectral será estimado pelo algoritmo de transformação rápida de Fourier, sendo considerados para fins de análise os componentes de alta (HF: 0,15 a 0,40Hz), baixa frequência (LF: 0,04 a0,15Hz) e a razão entre os componentes (LF/HF). Os valores dos diversos componentes serão calculados sob a forma de unidades normalizadas (n.u.), dividindo-se a potência de cada componente pela potência total, subtraída do valor do próprio componente e multiplicado por 100.

3.8 Análise estatística

O tamanho da amostra foi calculado pelo software GPower (versão 3.0.10, Universidade de Kiel, Kiel, Alemanha) com base em uma diferença de 6,7 mmHg para o ganho metaborreflexo (desvio padrão de 10 mm Hg). Assumindo potência de 80%, nível de significância de 5% e aumentando o tamanho da amostra em 50% devido às perdas de follow-up ao longo das 12 semanas do estudo, 22 pacientes em cada grupo foi determinado como necessário.

Para avaliação da atividade ergorreflexa, será considerada a média dos dois últimos minutos de recuperação obtido nos protocolos PECA e PE. Em seguida, será calculada a diferença entre PECA e PE. Este procedimento permitirá a avaliação do ganho metaborreflexo, ou seja, a resposta devido à atividade metaborreflexa. As diferenças entre os grupos serão avaliadas por meio de ANOVA fatorial de três entradas (grupo *vs* tempo *vs* treinamento) seguidas pelo teste post hoc de Tukey, quando apropriado. A análise estatística será realizada através de software comercialmente disponível (Stata 13.0), assumindo-se nível significativo estabelecido em $P < 0,05$ em todos os casos.

4 CRONOGRAMA

A seguir estão apresentadas as atividades que serão realizadas em cada um dos semestres durante a execução do projeto.

Quadro 1. Cronograma de execução

**Atividades/Quadrimestres**	**1° ano**		**2° ano**		**3° ano**		**4° ano**	
Atualização Bibliográfica	■	■	■	■	■	■	■	■
Obtenção da aprovação no comitê de ética	■							
Execução de testes piloto		■						
Intervenção (treinamento físico)			■	■	■	■		
Coleta e análise de dados			■	■	■	■		
Divulgação de resultados preliminares / Qualificação				■	■			
Submissão de artigos originais							■	■
Redação da tese e defesa							■	■

5 REFERÊNCIAS

1. Florindo, A.A., et al., *Validity and reliability of the Baecke questionnaire for the evaluation of habitual physical activity among people living with HIV/AIDS.* Cadernos de saude publica, 2006. **22**: p. 535-541.
2. Stroop, J.R., *Studies of interference in serial verbal reactions.* Journal of experimental psychology, 1935. **18**(6): p. 643.

6 ANEXO 1 – TERMO DE CONSENTIMENTO LIVRE E ESCLARECIDO

CÓDIGO DO PACIENTE ______________

FORMULÁRIO DE INFORMAÇÃO AO PACIENTE

"Documento elaborado por aplicação das recomendações da Declaração de Helsinki"

O Senhor está sendo convidado a participar de um estudo de pesquisa intitulado "RESPOSTAS HEMODINÂMICAS E AUTONÔMICAS À ATIVAÇÃO ERGORREFLEXA MUSCULAR EM PACIENTES VIVENDO COM HIV: EFEITOS DO TREINAMENTO FÍSICO" organizado pelo Laboratório de Atividade Física e Promoção da Saúde, no Instituto de Educação Física e Desportos da Universidade do Estado do Rio de Janeiro (UERJ) sob responsabilidade dos pesquisadores Juliana Borges e Gabriel Gama.

1. OBJETIVOS DO ESTUDO

Objetivo geral

Investigar a resposta autonômica e hemodinâmica durante ativação metaborreflexa e o impacto do treinamento físico nessa resposta em pessoas vivendo com HIV.

Objetivos específicos

- Verificar a resposta autonômica e hemodinâmica durante a ativação mecanorreflexa e o impacto do treinamento físico nessa resposta em pessoas vivendo com HIV;
- Verificar a função barorreflexa durante a ativação ergorreflexa e o impacto do treinamento nessa resposta em pessoas vivendo com HIV;
- Verificar o impacto do exercício sobre a modulação autonômica em pessoas vivendo com HIV;
- Comparar a resposta hemodinâmica e autonômica entre homens e mulheres com HIV à ativação do metaborreflexo muscular;
- Correlacionar a resposta hemodinâmica e autonômica à ativação do metaborreflexo muscular com indicadores de aptidão física dos pacientes.

2. PROCEDIMENTOS DO ESTUDO

Você será atendido no Laboratório de Atividade Física e Promoção da Saúde do Instituto de Educação Física e Desportos da UERJ (Endereço: Pavilhão João Lira Filho, 8º andar. Rua São Francisco Xavier 524, Maracanã) em dois dias não consecutivos por profissionais de educação física, os quais lhe explicarão os objetivos do presente estudo proposto e assinatura do Termo de Consentimento Livre e Esclarecido (TCLE).

Serão realizados os seguintes procedimentos:

Avaliações:

- Anamnese com medidas de peso e altura;
- Aplicação do questionário Baecke para determinação do nível de atividade física habitual;
- Verificação da modulação autonômica durante repouso, e durante a aplicação do teste de cores e palavras de Stroop através da variabilidade da frequência cardíaca;
- Realização do teste de força máximo para extensão unilateral de joelho.
- Aplicação do protocolo de avaliação ergorreflexa, que consiste na realização do exercício de extensão unilateral de joelho (cadeira extensora) de forma isométrica e passiva, com e sem a restrição circulatória através de um manguito de pressão posicionado no terço proximal da perna do avaliado;
- Verificação das respostas autonômicas, hemodinâmicas, e metabólicas ao protocolo de avaliação ergorreflexa, através da variabilidade da frequência cardíaca, da fotoplestimografia (similar ao eletrocardiograma) e da coleta de lactato sanguíneo, respectivamente.

Treinamento Físico:

Após a segunda visita para avalições, daremos início ao programa de treinamento físico que será composto por exercícios aeróbios, resistidos e de flexibilidade com duração total de 12 semanas, sendo realizado 3 vezes por semana.

3. BENEFÍCIOS E RISCOS POTENCIAIS

Alguns efeitos adversos, embora pouco frequentes, são descritos durante a realização de alguns exames e testes:

- Testes de força muscular máxima – o teste pode, em algumas pessoas, causar desconforto muscular tardio;
- Restrição circulatória – a restrição circulatória pode, em algumas pessoas, causar desconforto momentâneo (como câimbra). A presença de qualquer desconforto produzirá interrupção do teste imediatamente.
- Coleta de lactato sanguíneo – a coleta de lactato pode, em algumas pessoas, por se tratar de um procedimento minimamente invasivo, causar um leve desconforto momentâneo no dedo do voluntário.

Como benefício, será realizada uma análise de controle completa da pressão arterial, frequência cardíaca, dentre outras variáveis hemodinâmicas, de forma totalmente gratuita para o voluntário.

4. DESCONTINUAÇÃO VOLUNTÁRIA DO ESTUDO

Você poderá se recusar a participar da pesquisa, e mesmo que decida participar, você poderá se retirar do estudo no momento que assim desejar sem precisar dar explicações. Isto não afetará no tratamento, nem na relação médico-paciente com seu médico.

5. PROTEÇÃO DO PACIENTE E ÉTICA

Este estudo será realizado de acordo com os princípios estabelecidos na Declaração de Helsinki e emendas subsequentes (2000) e de acordo com as Boas Práticas Médicas (ICH/E6).

6. CONFIDENCIALIDADE

A confidencialidade do estudo está garantida pelo fato de que seu código como paciente (um número e iniciais) irão aparecer em todos os documentos escritos do estudo. As informações coletadas serão processadas por computador e poderão ser checadas pelas autoridades de saúde.

Se você tiver qualquer dúvida sobre este estudo, por favor entre em contato com o pesquisador responsável pelo estudo, Profa. Dra. Juliana Pereira Borges (LABSAU), no telefone (21) 2334-0775.

Pesquisador responsável pelo Projeto de Pesquisa
Profa. Dra. Juliana Borges (LABSAU)

Execução do Projeto de Pesquisa
Prof. Me. Gabriel da Silva Gama (LABSAU)

SESSÃO DO INVESTIGADOR (para ser preenchida pelo investigador)

Nome:..

Pessoa para Contato: Juliana Pereira Borges Número do telefone: (021) 2334-0775
Eu confirmo que tenha explicado o experimento em detalhes para o paciente. Eu informei a ele o formulário informativo e respondi todas as suas dúvidas relacionadas ao estudo.

Ass.:………………………………………………………………….. Data: ....

SESSÃO DO VOLUNTÁRIO (para ser preenchida pelo voluntário)

Nome:……………………………………………………………………………………………......................

Endereço:..

Eu recebi, li e entendi o formulário informativo para o estudo designado acima. Eu também recebi explicações adequadas sobre o estudo clínico, seus propósitos, riscos, meus direitos como paciente e o que terei de fazer e me submeter. Foram-me dadas todas as oportunidades de perguntar qualquer coisa antes de tomar qualquer decisão. Eu poderei necessitar de informações adicionais a qualquer momento do investigador. Eu sei que minha decisão de participar deste estudo só depende de mim mesmo, e que tenho o direito de mudar de ideia a qualquer momento durante o curso do estudo sem que isso venha afetar meu tratamento no futuro. Eu entendi que o experimento ou a minha participação nele podem ser interrompidas a qualquer momento pelo investigador. Eu também entendi que o acesso a informações relevantes sobre meus dados como paciente podem ser requeridas como parte do estudo e que dados coletados durante o estudo poderão ser checados pelas autoridades de saúde e por representantes do patrocinador de acordo com a legislação vigente.
Estou ciente de que meu anonimato será preservado com a mais estrita confidencialidade. Os dados registrados durante o estudo serão processados por computador e eu entendo que terei o direito, caso queira, de acesso aos dados computadorizados. Eu recebi uma cópia deste documento e fui informado que uma cópia será mantida confidencialmente pelo patrocinador do estudo. Sendo assim, eu dou meu consentimento para fazer parte deste estudo.

Assinatura:..

Datado pelo voluntário:………/…………./…...……

Universidade do Estado do Rio de Janeiro
Instituto de Educação Física e Desportos

EMENDA AO PROJETO DE PESQUISA ORIGINAL

RESPOSTAS HEMODINÂMICAS E AUTONÔMICAS À ATIVAÇÃO ERGORREFLEXA MUSCULAR EM PACIENTES VIVENDO COM HIV/AIDS: EFEITOS DO TREINAMENTO FÍSICO

Equipe de Pesquisa

Juliana Pereira Borges - Investigador Principal

Gabriel da Silva Gama - Investigador Associado

A presente solicitação de emenda se faz necessária devido a 3 modificações no protocolo do estudo constatadas como necessárias com base nos testes pilotos: 1) o protocolo de exercício passivo (PE) para avaliação mecanorreflexo foi retirado, e por esse motivo, somente será necessária a realização de uma visita ao laboratório, ao invés de duas visitas como originalmente apresentado; 2) o exercício de extensão do joelho foi substituído pelo exercício de preensão manual; e 3) a avaliação do metaborreflexo muscular passará a ser realizado sem e com a aplicação de uma pomada à base de capsaicina, que é comercialmente vendida para o tratamento de osteoartrite, com o intuito de amplificar a ativação do metaborreflexo muscular antes de PECA.
