# Supplementary material for: The effects of exercise training on autonomic and hemodynamic responses to muscle metaboreflex in people living with HIV/AIDS: A randomized clinical trial protocol
Source: PLoS One. 2022 Mar 18;17(3):e0265516. doi: 10.1371/journal.pone.0265516 (PMC8932586; doi:10.1371/journal.pone.0265516)
Supplement: Attachment — Submitted filename: Comments to the authors 2022.docx [file pone.0265516.s004.docx]

**The effects of exercise training on autonomic and hemodynamic responses to muscle metaboreflex in people living with HIV/AIDS: a randomized clinical trial protocol.**

Gabriel Gama^1,2^, Marcus Vinicius dos Santos Rangel^1,2^, Vanessa Cunha de Oliveira Coelho^1^, Gabriela Andrade Paz^1,2^, Catarina Vieira Branco de Matos^1^, Bárbara Pinheiro Silva^1^, Gabriella de Oliveira Lopes^1^, Karynne Grutter Lopes^1,3^, Paulo Farinatti^1,2^, Juliana Pereira Borges^1,2*^

^1^Laboratory of Physical Activity and Health Promotion, Institute of Physical Education and Sports, University of Rio de Janeiro State, Rio de Janeiro, RJ, Brazil. ^2^Graduate Program in Exercise and Sports Sciences, University of Rio de Janeiro State, Rio de Janeiro, RJ, Brazil. ^3^Graduate Program in Clinical and Experimental Physiopathology, Faculty of Medical Sciences, University of Rio de Janeiro State, Rio de Janeiro, RJ, Brazil.

**Running title:** HIV infection, exercise pressor reflex and training

**Word Count:** Main document: 5434 words; Abstract: 273 words; Figures: 4

*** Corresponding author:** Juliana P. Borges, PhD (ORCID 0000-0002-3581-7251**).** Laboratory for Physical Activity and Health Promotion, University of Rio de Janeiro State. Rua São Francisco Xavier, 524, Pavilhão João Lyra Filho, sala 8121F, Maracanã, Rio de Janeiro, RJ, CEP 20550-013. Phone: (+55-21) 2334-0775; E-mail: [julipborges@gmail.com](mailto:julipborges@gmail.com)

**Abstract**

**Background**: People living with HIV (PLHIV) present impaired muscle metaboreflex, which may lead to exercise intolerance and increased cardiovascular risk. The muscle metaboreflex adaptations to exercise training in these patients are unknown. The present study aims to investigate the effects of a supervised multimodal exercise training on autonomic and hemodynamic responses to muscle metaboreflex activation in PLHIV. **Methods and design**: In this randomized clinical trial protocol, PLHIV aged 30-50 years will be randomly assigned at a ratio of 1:1 into an intervention or a control group. The intervention group will perform exercise training (3x/week during 12 weeks) and the control group will remain physically inactive. A reference group composed of HIV-uninfected individuals will be included. Primary outcomes will be heart rate variability parameters and blood pressure assessed by a heart rate monitor and a digital sphygmomanometer, respectively; during a resting period, mental stress, and activation of muscle metaboreflex. Mental stress will be induced by the Stroop Color-Word test and muscle metaboreflex will be activated through a post-exercise circulatory arrest (PECA) protocol, being the latter performed without and with the application of a capsaicin-based analgesic balm in the exercised limb. Secondary outcomes will be heart rate, peripheral vascular resistance, stroke volume, cardiac output, and blood lactate. The intervention and control groups of PLHIV will be evaluated at baseline and after the intervention, while the HIV-uninfected reference group only at baseline. **Discussion:** The findings of the present study may help to elucidate the muscle metaboreflex adaptations to exercise training in PLHIV.

**Trial registration:** This study will be performed at University of Rio de Janeiro State following registration at ClinicalTrials.gov as NCT04512456 on August 13, 2020.

**Keywords:** AIDS; exercise training; ergoreflex; muscle ergoreflex; exercise pressor reflex.

**INTRODUCTION**

The Acquired Immunodeficiency syndrome (AIDS) is a chronic, potentially life-threatening condition caused by the Human Immunodeficiency Virus (HIV). Globally, 690,000 people have died from AIDS-related illnesses in 2019 and 1.7 million new infections occurred in that year. Access to combined antiretroviral therapy (cART) has prevented around 12.1 million AIDS-related deaths since 2010, so that 38 million people are currently living with HIV (1).

Despite the increase in life expectancy, the combination of prolonged HIV infection and cART treatment may damage several physiological systems, thereby increasing the risk of non-AIDS related diseases (2, 3). A recent review (3) revealed that cardiometabolic diseases consistently appeared among the most reported comorbidities in people living with HIV (PLHIV), whose rates of myocardial infarction, heart failure, pulmonary hypertension, sudden cardiac death and stroke are higher in comparison with uninfected subjects (4). Additionally, increased sympathetic activity, which is a marker of poor cardiovascular prognosis (5) is often found among PLHIV at rest (6-8) and after physical exercise (9).

During exercise, hemodynamic function mediated by autonomic activity is mainly regulated by the exercise pressor reflex (EPR) (10, 11), which responds to muscle afferent signals from type III (mechanoreflex) and IV (metaboreflex) nerve endings (12-15). Those afferent neurons are sensitive to mechanical stimuli and metabolite accumulation, respectively (16, 17). The EPR increases the sympathetic stimulation to both heart and blood vessels, in order to meet the augmented metabolic demand of working muscles (18). If muscle mechano- or metaboreflex is blunted, the increase in blood pressure (BP) may not be enough to assure appropriate muscle blood flow and oxygen supply, leading to premature fatigue and exercise intolerance (19, 20). Considering that abnormal skeletal muscle reflexes play a significant role in limiting the exercise tolerance, as well as in increasing the cardiovascular risk during or after exercise (18), investigations on strategies capable to counteract those impairments are clinically relevant.

Exercise training seems to improve sympathetic activity control and/or abnormal cardiovascular responses to muscle metaboreflex activation in several chronic conditions (21-24). Although very little information is available on the potential mechanisms by which exercise training favors the muscle metaboreflex, they seem to include a wide spectrum of adaptations, as increased muscle perfusion with lower metabolite accumulation, greater antioxidant capacity, and changes in the expression of receptors evoking metaboreflex (i.e., the transient receptor potential vanilloid receptor 1 – TRPv1) (25). Individuals with impaired metaboreflex have deficiencies in one or more of those mechanisms, and therefore might be more responsive to exercise training than healthy individuals (25). This is the case of PLHIV, who often present lactic acidosis (26) and attenuated pressor response to metaboreceptor stimulation (27).

Despite this, we could not find research investigating muscle metaboreflex adaptations and its underlying mechanisms to exercise training in PLHIV. This would be nonetheless useful to determine the potential impact of exercise training on mechanisms controlling autonomic modulation during exercise, which have prognostic value (28). Thus, the aim of the present study is to describe the experimental protocol of a randomized controlled trial designed to investigate the effects of a multimodal supervised exercise program on autonomic and hemodynamic responses to muscle metaboreflex activation in PLHIV. In addition, potential mechanisms of exercise training adaptations in the muscle metaboreflex will be analyzed by assessing blood lactate concentration and activating TRPv1. We will test the hypothesis that a multimodal exercise training would be able to increase the neurocardiovascular responses during metaboreflex activation by reducing blood lactate levels at rest and increasing TRPv1 sensitivity.

**MATERIALS AND METHODS**

**Study Design**

This single-center randomized clinical trial will be performed at the Laboratory of Physical Activity and Health Promotion (LABSAU) at the University of Rio de Janeiro State (RJ, Brazil), where a supervised exercise program particularly designed for PLHIV is held since 2005. PLHIV will be recruited in the outpatient clinic of the University hospital, and uninfected volunteers matched for age, sex and body mass will be randomly recruited from the staff of the same institution to compose a reference group. All subjects will receive oral and written instructions about the study risks and benefits and will be included in the study only after giving written informed consent. Recruitment, pre-participation screening and data collection will occur between January 2022 and December 2023. We believe that results will be available for analysis in 2024.

The trial was registered at ClinicalTrials.gov (registration number: NCT04512456, date of registration: August 13, 2020) and approved by the Ethics Committee of the Pedro Ernesto University Hospital (approval number 41955620.2.0000.5259, opinion number 4.520.721, approved in February 2, 2021; supplementary information file S1). The study complies with the SPIRIT 2013 recommendations (Standard Protocol Items: Recommendations for International Trials) (supporting information file S2) (29). The Figure 1 depicts the overall schedule and time commitment for trial participants (29).

Figure 1. Schedule of enrolment, interventions, and assessments. -*t*_1_, enrolment week 0; *t*_0_, allocation and baseline week; *t*_3_, post-intervention (week 12).

**Participants and recruitment**

Eligible PLHIV will be men and women aged 30- to 50 years, diagnosed with HIV/AIDS (30), but asymptomatic and free from opportunist infections. Exclusion criteria will be: a) use of cART for less than 3 years; b) regular physical exercise (≥ 3 days/week during 30 min) in the last 6 months; c) current or past diagnosis of hypertension, coronary artery disease, ischemic disease, pulmonary disease, diabetes mellitus, Chagas disease, tuberculosis, or heart failure; d) malnutrition; e) use of antidepressant or antihypertensive medication, and f) smoking habit. Uninfected controls will be screened for items b, c, d, e, and f.

PLHIV will be randomized through a random code generator software (www.randomization.com) at a ratio of 1:1 into intervention or control groups. Therefore, the experiment will have three groups: a) PLHIV under training (PLHIV-T), which will enroll in the multimodal exercise training during 12 weeks; b) PLHIV controls (PLHIV-C), which will remain in clinical care and shall not to change their physical activity behavior; c) Uninfected controls (CONT), which shall not change their physical activity behavior. For ethical reasons, the PLHIV-C group will have the possibility to undertake the exercise training intervention once the trial ends. Outcomes in PLHIV-T and PLHIV-C will be assessed before randomization at baseline and after intervention, whereas in CONT only at baseline to provide reference values. The study flowchart is presented in Figure 2.

Figure 2. Study flowchart.

**Experimental Design**

Assessments will be undertaken in a single visit, as shown in Figure 3. All procedures will take place at the same time of the day (9–11 am) to minimize circadian effects, in a quiet temperature-controlled environment (21–23 ^o^C). Participants will be instructed to fast for 2 h, to avoid physical exercises in the 48 h, and caffeine or alcohol in the 12 h prior to experimental sessions.

After a general medical examination and anthropometric measurements, participants will remain 30 min at rest in a supine position before performing a mental stress task and handgrip strength test. The mental stress task will provide information about the integrity of pathways of stress-induced sympathetic responses mediated by the central command. Maximal voluntary contraction (MVC) assessed by the handgrip test will be used to determine the loads applied during the metaboreflex activation protocol, and to detect possible strength differences between groups at baseline and after intervention. Subsequently, the muscle metaboreflex will be activated using a post-exercise circulatory arrest (PECA) protocol, during which blood lactate will be assessed. Then, a capsaicin-based analgesic balm (0.1%, FarmaSite, Itaúna, Brazil) will be applied over the exercising arm to stimulate the metabolically sensitive muscle afferents via TRPv1 receptors (31), and after 30 min of topical application, PECA will be repeated.

Primary outcomes will be BP and heart rate variability (HRV) indices reflecting autonomic modulation. Secondary outcomes will be the heart rate (HR), peripheral vascular resistance (PVR), stroke volume (SV), cardiac output (CO), blood lactate concentration, anthropometric markers, and handgrip MVC.

Figure 3. Experimental design.

**Intervention**

Patients included in the intervention arm will participate of an exercise training program during 12 weeks, consisting of 60-min sessions performed 3 days/week and including aerobic, resistance, and flexibility exercises, as described elsewhere (32). Treadmill or cycle ergometer aerobic exercise will last 30 min with an intensity range corresponding to 50–80% of the heart rate reserve. Resistance training will consist of 20 min of exercises for the major muscle groups of the upper and lower limbs (8–10 single and multi-joint exercises with free weights and machines), performed with 2–3 sets of 10–12 maximum repetitions (RM) and load corresponding to 80–90% of 10 RM. At the end of the sessions, the participants will perform a 10-min static stretching routine involving major joints (2 sets of 6- to 8 exercises, holding the maximal range of motion for 30-s).

Heart rate will be monitored to ensure proper aerobic exercise intensity (Polar H10; Polar Electro^TM^, Kempele, Finland). All training sessions will be performed indoors (21-23 ^o^C), always in the morning and under the supervision of the research staff. The participants will be instructed on the importance of clinical follow-up and the benefits of regular physical activity to improve adherence to intervention.

**Mental stress test**

To investigate the integrity of muscle metaboreflex efferent pathways, a mental stress assessment will be performed using the Stroop Color-Word test (33), which has been widely used for stress-induced sympathetic effects mediated by the central command, regardless of the muscle metaboreflex activation (34). The test will be administered for 5 min by a single researcher that will display slides changing every 2 s, showing color names written in different colors. The volunteers will identify the word colors irrespective of the written information. Changes from rest in autonomic and cardiovascular outcomes will be recorded as outcomes.

**Muscle metaboreflex activity assessment**

Muscle metaboreflex will be activated by a PECA protocol, which has been shown to trap muscle metabolites in the exercising limb and to maintain stimulation of group IV muscle afferents without the activity of the central command and mechanoreflex (35). PECA will consist of 13 min, as follows: 5 min of rest, 2 min of isometric handgrip exercise with the dominant arm with a load corresponding to 40% of MVC, 3 min of vascular occlusion, followed by 3 min of recovery. Vascular occlusion will be applied in the exercised limb by inflating a cuff at 240 mmHg 5 s before the end of exercise, and then deflating it in the 3-min recovery. During PECA, the volunteers will be in a sitting position, and shall avoid performing the Valsalva maneuver and any movement, except for the handgrip. Changes from rest during PECA will be calculated for autonomic and cardiovascular outcomes.

Prior to PECA, a brief familiarization with the handgrip exercise will be allowed. Handgrip force exerted will be placed in front of the subjects to guide the contraction intensity. Vascular occlusion in PECA will be applied using a nylon cuff size 11 x 85 cm connected to a pneumatic cuff inflator Hokanson TD312 (D. E. Hokanson^TM^ Inc, Bellevue, Washington, USA) placed around the proximal third of the dominant arm. A vascular Doppler probe (DV 610B Medmega Industria de Equipamentos Medicos, Franca, SP, Brazil) will be placed over the brachial artery (dominant arm) to capture the auscultatory pulse and confirm the vascular occlusion during the PECA protocol. The Figure 4 details the PECA protocol.

Figure 4. Post-exercise circulatory arrest (PECA) protocol used during muscle metaboreflex activity assessment.

**Primary outcomes**

*Blood pressure*

Systolic and diastolic BP will be assessed in the non-dominant arm using a digital sphygmomanometer (Omron HEM - 7113, Kyoto, Japan) positioned around the proximal third of the non-dominant arm. Data will be assessed at rest, and every minute during mental stress and muscle metaboreflex assessments.

*Autonomic indices*

The cardiac autonomic modulation will be assessed through HRV. Beat-to-beat HR will be continuously recorded using a heart rate monitor (Polar V800; Polar^TM^ Electro, Kempele, Finland) (36){Gilgen-Ammann, 2019 #35} at rest, during the mental stress task, and muscle metaboreflex activation protocol. The signals will be transferred to the Polar Precision Performance Software (Polar Electro, Kempele, Finland). After replacing the non-sinus beat by interpolated data derived from adjacent normal RR intervals, times series data will be exported to Kubios^TM^ HRV program for analysis (Version 3.4, University of Kuopio, Kuopio, Finland).

Time domain variables will be: mean normal RR intervals (RRi), standard deviation of normal NN intervals (SDNN), square root of the mean squared successive differences from adjacent RRi (rMSSD), and percentage number of pairs of adjacent RRi differing by more than 50 ms from previous RRi (pNN50). SDNN, rMSSD, and pNN50 are acknowledged to reflect the parasympathetic modulation within short periods (37). Spectral analysis will be performed by the Fast Fourier transform method, with frequency bands corresponding to low-frequency (LF: 0.04–0.15 Hz), high-frequency (HF: 0.15–0.40 Hz), and total power (TP; meaning LF plus HF) (37). The LF and HF reflect a predominance of combined sympathetic-parasympathetic and isolated parasympathetic modulation, respectively (37), while LF: HF ratio will be adopted as a marker of sympathovagal balance.

**Secondary outcomes**

*Hemodynamic parameters*

CO will be continuously and non-invasively measured at rest, during mental stress and muscle metaboreflex activity assessment by the indirect Fick method of CO_2_ rebreathing (38) using a metabolic cart (Ultima CardiO_2_ System, Medical Graphics Corporation, Minnesota, USA). In this method, individuals breathe spontaneously until a steady CO_2_ production occurs. This procedure is followed by the rebreathing of a mixed gas with a high CO_2_ concentration (8–10%) and 35% O_2_ until equilibrium is achieved (maximal of 15 s). At this moment, CO is calculated by the Fick formula. PVR will be calculated by the quotient between mean BP and CO, and SV by the quotient between CO and HR.

*Blood lactate concentration*

Blood lactate will be measured to verify the metabolic stress induced by PECA. With this purpose, the fingertip of the exercising arm will be punctured with an automatic lancet and 25 µl of capillary blood sample will be collected during PECA, at minutes 5, 7 and 10. The blood sample will be transferred to a microtube containing 50 µl of 1% sodium fluoride, and blood lactate concentration will be determined by the YSL 2700 analyzer (Yellow Spring^TM^ Co., Yellow Springs, Ohio, USA).

*Anthropometry*

Height and body mass will be assessed by a digital scale (Filizola^TM^, Sao Paulo, SP, Brazil), and wall-mounted stadiometer (Sanny^TM^, Sao Paulo, SP, Brazil), respectively. The BMI will be calculated (Kg/m^2^).

*Handgrip maximal strength test*

Handgrip MVC will be assessed using a hydraulic handgrip dynamometer (Saehan, SH5001, Holliston, MA, USA). Strength measurements will be performed with participants in a sitting position, arm along the body, and elbow in flexion (dominant arm). The volunteers will make three attempts of 5 s interspersed with 2-min intervals, and the highest value will be recorded as result.

**Sample size calculation and statistical analysis**

Sample size was calculated using GPower 3.1.9.4 (Universität Kiel, Kiel, Germany) to detect longitudinal differences between groups in systolic BP response due to metaboreflex gain. Considering a between-group difference of 3.5 mmHg (standard deviation of 4.6 mmHg) (27), 80% power and 5% significance level, and increasing the sample size by 50% to account for losses to follow up, a total of 14 participants in each group was calculated as necessary.

Descriptive analysis will consist of mean and standard deviation for continuous variables and percentage for categorical variables. Data normality will be checked by the Shapiro Wilk test, and data will be presented as mean and standard deviation or percentage, whenever appropriate. A cross-sectional comparison between PLHIV-T, PLHIV-C and CONT will be performed at baseline by means of one-way ANOVA (continuous) or chi-squared test (categorical).

Longitudinal effects of exercise training on primary and secondary outcomes will be evaluated through linear mixed models (LMM) including treatment (PLHIV-T or PLHIV-C) and time as fixed effects, and group *vs.* time interaction. LMM is an intention-to-treat analysis as it includes all observations of each one of the participants, regardless of losses to follow-up or noncompliance to exercise protocol. Residual plots of all models will be examined and the likelihood-ratio test will be used to compare and select random intercept or random slope models. Data analysis will be performed using Stata 13.0 (StataCorp, College Station, TX, USA), and in all cases the statistical significance will be set at *P* ≤ 0.05.

**Potential harms**

In the event of any clinical intercurrence during the training sessions or experimental protocol, the participants will be referred to the University Hospital for medical care. Any adverse clinical event will be reported to the ethics committee.

**Data management, accessibility, and dissemination policy**

A trained research assistant will be responsible for including all primary and secondary outcomes in an Excel spreadsheet in the proper format for analysis, which will be checked by the principal investigator. Unnamed data collected and research results will be deposited in the cloud (Dropbox) and may be consulted after permission granted by the principal investigator. All personal data will be properly stored in folders that will be kept confidential, and will not be passed on to any third party without prior consent from the participants. At the study end, each participant will receive a full report with the results of their assessments. The results of the study will be publicized in scientific congresses and journals.

**Masking and blinding**

The behavioral feature of intervention (exercise training) precludes the blinding of the patients. On the other hand, data analysis will be performed by a single trained researcher, blinded for the group allocation and use or not of capsaicin-based analgesic balm in data collected during PECA.

**DISCUSSION**

The findings of the present study may help to elucidate the role of exercise training to produce adaptations in autonomic and hemodynamic responses induced by muscle metaboreflex in PLHIV. Prior research has demonstrated that PLHIV present abnormal pressor response during muscle metaboreceptor stimulation (27) and autonomic dysfunction at rest (39) and after exercise (9). When group IV muscle afferent feedback is impaired, the increase in BP may not be enough to assure an appropriate muscle blood flow and oxygen supply, leading to premature fatigue and exercise intolerance (20), as previously reported in PLHIV (40).

Most studies addressing the effects of exercise training in PLHIV have focused on physical fitness, body composition, immune function, mental health or lipid profile (41-43), whereas the neurocardiovascular function of these patients has gained little attention. To the best of our knowledge, there are only two clinical trials investigating the role of exercise training on the autonomic function in PLHIV (44, 45). Quiles et al. (44) have recently demonstrated that an 8-week aerobic exercise program improved the overall autonomic function of these patients, while Pedro et al. (45) did not observe changes in HRV after 16 weeks of concurrent training. Additionally, we could not locate studies investigating the role of exercise training on the muscle metaboreflex among these patients.

In the present protocol, the muscle metaboreflex will be activated by PECA, which is a non-invasive and non-pharmacological method widely used to isolate the metabolic component of ergoreflex, by arresting blood flow of the exercising muscles with a suprasystolic level cuff occlusion that traps the metabolic by-products of exercise within the muscles after the cessation of exercise (46-48). Of note, PECA will be originally employed in combination to the topical application of a capsaicin-based analgesic balm over the exercising arm. The capsaicin is an active component of chili peppers targeting the TRPv1, which are important mediators of the metabolic stimulation of EPR (31). Evidence has recently shown that a topical application of a capsaicin-based analgesic balm prior to the test attenuated the pressor and muscle sympathetic nervous activity responses to PECA (31). However, capsaicin-based analgesic balms to assess exercise training-related adaptations on muscle metaboreflex have never been used. Another important aspect of this study is the multimodal characteristic of the exercise training protocol (e.g., aerobic and resistance exercises), as most studies using this modality of training have found benefits in EPR among other populations (21-23, 49), while those including only resistance training failed to observed similar adaptations (50-53).

The present study protocol has some limitations. Firstly, autonomic modulation will be assessed by HRV. Although HRV has the advantage of being a simple, non-invasive method capable of assessing dynamic changes in the autonomic control of heart rate (54), more objective measurements of muscle sympathetic outflow, such as microneurography would provide more specific and direct information. Moreover, it will not be possible to control the diet and sleep habits of participants. Despite this, the present study might extend the current knowledge by demonstrating whether a supervised multimodal exercise training would be capable to improve the muscle metaboreflex among PLHIV. In addition, we also intend to provide new insights into the mechanisms potentially underlying the exercise-induced changes in the muscle metaboreflex, by assessing the influence of TRPv1 receptors, blood lactate concentration, and hemodynamic outcomes (e.g., HR, SV, CO, and PVR).

**Author contributions statement**

The conception and design of the study were made by GG and JB and discussed with the other authors. GG, PF, MVSR, GAP, CVBM, BPS, KGL and JB drafted and revised the study protocol. GG, MVSR, VCOC, CVBM, BPS and GOL will collect data, while PF and JPB will perform statistical analyzes. JPB will be the principal investigator supervising all the experiment. All authors read and approved the final version of this study protocol.

**Conflict of interest statement**

The authors declare that the research is conducted without any commercial or financial relationships that could be considered a potential conflict of interest.

**REFERENCES**

1.	UNAIDS. Global HIV & AIDS statistics — 2020 fact sheet. January 4, 2021. Retrieved from <https://www.unaids.org/en/resources/fact-sheet>. [

2.	Panel-of-Experts-from-the-Metabolic-Disorders-and-Comorbilities Study-Group-(GEAM), Aids-Study-Group-(GeSIDA), National-Aids-Plan-(PNS). Executive summary of the consensus document on metabolic disorders and cardiovascular risk in patients with HIV infection. Enferm Infecc Microbiol Clin. 2019;37(1):50-5.

3.	De Francesco D, Sabin CA, Reiss P. Multimorbidity patterns in people with HIV. Curr Opin HIV AIDS. 2020;15(2):110-7.

4.	Feinstein MJ, Hsue PY, Benjamin LA, Bloomfield GS, Currier JS, Freiberg MS, et al. Characteristics, Prevention, and Management of Cardiovascular Disease in People Living With HIV: A Scientific Statement From the American Heart Association. Circulation. 2019;140(2):e98-e124.

5.	Schwartz PJ, La Rovere MT, Vanoli E. Autonomic nervous system and sudden cardiac death. Experimental basis and clinical observations for post-myocardial infarction risk stratification. Circulation. 1992;85(1 Suppl):I77-91.

6.	Spierer DK, DeMeersman RE, Kleinfeld J, McPherson E, Fullilove RE, Alba A, et al. Exercise training improves cardiovascular and autonomic profiles in HIV. Clinical autonomic research : official journal of the Clinical Autonomic Research Society. 2007;17(6):341-8.

7.	Sakhuja A, Goyal A, Jaryal AK, Wig N, Vajpayee M, Kumar A, et al. Heart rate variability and autonomic function tests in HIV positive individuals in India. Clinical autonomic research : official journal of the Clinical Autonomic Research Society. 2007;17(3):193-6.

8.	Quiles N, Garber C, Ciccolo J. Resting Autonomic Function in Active and Insufficiently Active People Living with HIV. International journal of sports medicine. 2018;39(1):73-8.

9.	Borges J, Soares P, Farinatti P. Autonomic modulation following exercise is impaired in HIV patients. International journal of sports medicine. 2012;33(4):320-4.

10.	Nobrega AC, Araujo CG. Heart rate transient at the onset of active and passive dynamic exercise. Med Sci Sports Exerc. 1993;25(1):37-41.

11.	Chen CY, Bonham AC. Postexercise hypotension: central mechanisms. Exerc Sport Sci Rev. 2010;38(3):122-7.

12.	Alam M, Smirk FH. Observations in man upon a blood pressure raising reflex arising from the voluntary muscles. The Journal of physiology. 1937;89(4):372-83.

13.	Smith SA, Mammen PP, Mitchell JH, Garry MG. Role of the exercise pressor reflex in rats with dilated cardiomyopathy. Circulation. 2003;108(9):1126-32.

14.	Fisher JP, Adlan AM, Shantsila A, Secher JF, Sorensen H, Secher NH. Muscle metaboreflex and autonomic regulation of heart rate in humans. The Journal of physiology. 2013;591(15):3777-88.

15.	Williamson JW, Fadel PJ, Mitchell JH. New insights into central cardiovascular control during exercise in humans: a central command update. Experimental physiology. 2006;91(1):51-8.

16.	Kaufman MP, Hayes SG, Adreani CM, Pickar JG. Discharge properties of group III and IV muscle afferents. Sensorimotor control of movement and posture: Springer; 2002. p. 25-32.

17.	Mark AL, Victor RG, Nerhed C, Wallin BG. Microneurographic studies of the mechanisms of sympathetic nerve responses to static exercise in humans. Circulation research. 1985;57(3):461-9.

18.	Murphy MN, Mizuno M, Mitchell JH, Smith SA. Cardiovascular regulation by skeletal muscle reflexes in health and disease. Am J Physiol Heart Circ Physiol. 2011;301(4):H1191-204.

19.	Amann M, Sidhu SK, Weavil JC, Mangum TS, Venturelli M. Autonomic responses to exercise: group III/IV muscle afferents and fatigue. Autonomic neuroscience : basic & clinical. 2015;188:19-23.

20.	Amann M, Wan HY, Thurston TS, Georgescu VP, Weavil JC. On the Influence of Group III/IV Muscle Afferent Feedback on Endurance Exercise Performance. Exerc Sport Sci Rev. 2020;48(4):209-16.

21.	Antunes-Correa LM, Nobre TS, Groehs RV, Alves MJ, Fernandes T, Couto GK, et al. Molecular basis for the improvement in muscle metaboreflex and mechanoreflex control in exercise-trained humans with chronic heart failure. Am J Physiol Heart Circ Physiol. 2014;307(11):H1655-66.

22.	Guerra RS, Goya TT, Silva RF, Lima MF, Barbosa ERF, Alves M, et al. Exercise Training Increases Metaboreflex Control in Patients with Obstructive Sleep Apnea. Med Sci Sports Exerc. 2019;51(3):426-35.

23.	Trombetta IC, Batalha LT, Rondon MU, Laterza MC, Kuniyoshi FH, Gowdak MM, et al. Weight loss improves neurovascular and muscle metaboreflex control in obesity. Am J Physiol Heart Circ Physiol. 2003;285(3):H974-82.

24.	Milia R, Roberto S, Marongiu E, Olla S, Sanna I, Angius L, et al. Improvement in hemodynamic responses to metaboreflex activation after one year of training in spinal cord injured humans. BioMed research international. 2014;2014:893468.

25.	Gama G, Farinatti P, Rangel M, Mira PAC, Laterza MC, Crisafulli A, et al. Muscle metaboreflex adaptations to exercise training in health and disease. European journal of applied physiology. 2021.

26.	Thet D, Siritientong T. Antiretroviral Therapy-Associated Metabolic Complications: Review of the Recent Studies. HIV AIDS (Auckl). 2020;12:507-24.

27.	Gama G, Farinatti P, Crisafulli A, Borges J. Blood Pressure Response to Muscle Metaboreflex Activation is Impaired in Men Living with HIV. International journal of sports medicine. 2020.

28.	Michael S, Graham KS, Davis GMO. Cardiac Autonomic Responses during Exercise and Post-exercise Recovery Using Heart Rate Variability and Systolic Time Intervals-A Review. Frontiers in physiology. 2017;8:301.

29.	Chan A-W, Tetzlaff JM, Gøtzsche PC, Altman DG, Mann H, Berlin JA, et al. SPIRIT 2013 explanation and elaboration: guidance for protocols of clinical trials. BMJ : British Medical Journal. 2013;346:e7586.

30.	From the Centers for Disease Control and prevention. 1993 revised classification system for HIV infection and expanded surveillance case definition for AIDS among adolescents and adults. Jama. 1993;269(4):460.

31.	Vianna LC, Fernandes IA, Barbosa TC, Teixeira AL, Nobrega ACL. Capsaicin-based analgesic balm attenuates the skeletal muscle metaboreflex in healthy humans. Journal of applied physiology. 2018;125(2):362-8.

32.	Lopes GO, Farinatti P, Lopes KG, Medeiros-Lima DJ, Matsuura C, Oliveira RB, et al. Increased vascular function and superoxide dismutase activity in physically active vs inactive adults living with HIV. Scand J Med Sci Sports. 2019;29(1):25-33.

33.	Scarpina F, Tagini S. The Stroop Color and Word Test. Front Psychol. 2017;8:557.

34.	Tulen JH, Moleman P, van Steenis HG, Boomsma F. Characterization of stress reactions to the Stroop Color Word Test. Pharmacol Biochem Behav. 1989;32(1):9-15.

35.	Fisher JP, Young CN, Fadel PJ. Autonomic adjustments to exercise in humans. Compr Physiol. 2015;5(2):475-512.

36.	Gilgen-Ammann R, Schweizer T, Wyss T. RR interval signal quality of a heart rate monitor and an ECG Holter at rest and during exercise. European journal of applied physiology. 2019;119(7):1525-32.

37.	Heart rate variability. Standards of measurement, physiological interpretation, and clinical use. Task Force of the European Society of Cardiology and the North American Society of Pacing and Electrophysiology. Eur Heart J. 1996;17(3):354-81.

38.	Jones NL, Campbell EJ, McHardy GJ, Higgs BE, Clode M. The estimation of carbon dioxide pressure of mixed venous blood during exercise. Clin Sci. 1967;32(2):311-27.

39.	McIntosh RC. A meta-analysis of HIV and heart rate variability in the era of antiretroviral therapy. Clinical autonomic research : official journal of the Clinical Autonomic Research Society. 2016;26(4):287-94.

40.	Thoni GJ, Schuster I, Walther G, Nottin S, Vinet A, Boccara F, et al. Silent cardiac dysfunction and exercise intolerance in HIV+ men receiving combined antiretroviral therapies. Aids. 2008;22(18):2537-40.

41.	Gomes-Neto M, Conceicao CS, Oliveira Carvalho V, Brites C. A systematic review of the effects of different types of therapeutic exercise on physiologic and functional measurements in patients with HIV/AIDS. Clinics. 2013;68(8):1157-67.

42.	Dianatinasab M, Ghahri S, Dianatinasab A, Amanat S, Fararouei M. Effects of Exercise on the Immune Function, Quality of Life, and Mental Health in HIV/AIDS Individuals. Advances in experimental medicine and biology. 2020;1228:411-21.

43.	Zanetti HR, da Cruz LG, Lourenco CL, Ribeiro GC, Ferreira de Jesus Leite MA, Neves FF, et al. Nonlinear Resistance Training Enhances the Lipid Profile and Reduces Inflammation Marker in People Living With HIV: A Randomized Clinical Trial. Journal of physical activity & health. 2016;13(7):765-70.

44.	Quiles N, Taylor B, Ortiz A. Effectiveness of an 8-Week Aerobic Exercise Program on Autonomic Function in People Living with HIV Taking Anti-Retroviral Therapy: A Pilot Randomized Controlled Trial. AIDS Res Hum Retroviruses. 2020;36(4):283-90.

45.	Pedro RE, Guariglia DA, Okuno NM, Deminice R, Peres SB, Moraes SM. Effects of 16 Weeks of Concurrent Training on Resting Heart Rate Variability and Cardiorespiratory Fitness in People Living With HIV/AIDS Using Antiretroviral Therapy: A Randomized Clinical Trial. J Strength Cond Res. 2016;30(12):3494-502.

46.	Teixeira AL, Fernandes IA, Vianna LC. Cardiovascular Control During Exercise: The Connectivity of Skeletal Muscle Afferents to the Brain. Exerc Sport Sci Rev. 2020;48(2):83-91.

47.	Crisafulli A. The Impact of Cardiovascular Diseases on Cardiovascular Regulation During Exercise in Humans: Studies on Metaboreflex Activation Elicited by the Post-exercise Muscle Ischemia Method. Current cardiology reviews. 2017;13(4):293-300.

48.	Boushel R. Muscle metaboreflex control of the circulation during exercise. Acta physiologica. 2010;199(4):367-83.

49.	Notarius CF, Millar PJ, Keir DA, Murai H, Haruki N, O'Donnell E, et al. Training heart failure patients with reduced ejection fraction attenuates muscle sympathetic nerve activation during mild dynamic exercise. American journal of physiology Regulatory, integrative and comparative physiology. 2019;317(4):R503-R12.

50.	Ray CA, Carrasco DI. Isometric handgrip training reduces arterial pressure at rest without changes in sympathetic nerve activity. Am J Physiol Heart Circ Physiol. 2000;279(1):H245-9.

51.	Fisher WJ, White MJ. Training-induced adaptations in the central command and peripheral reflex components of the pressor response to isometric exercise of the human triceps surae. The Journal of physiology. 1999;520 Pt 2:621-8.

52.	Saito M, Iwase S, Hachiya T. Resistance exercise training enhances sympathetic nerve activity during fatigue-inducing isometric handgrip trials. European journal of applied physiology. 2009;105(2):225-34.

53.	Crisafulli A, de Farias RR, Farinatti P, Lopes KG, Milia R, Sainas G, et al. Blood Flow Restriction Training Reduces Blood Pressure During Exercise Without Affecting Metaboreflex Activity. Frontiers in physiology. 2018;9:1736.

54.	Johnston BW, Barrett-Jolley R, Krige A, Welters ID. Heart rate variability: Measurement and emerging use in critical care medicine. Journal of the Intensive Care Society. 2020;21(2):148-57.

**Funding**

The project will be funded by the Carlos Chagas Filho Foundation for the Research Support in the State of Rio de Janeiro (FAPERJ), grants E-26/010.100935/2018 and E-26/202.720/2019 recipient JB) (supplemental file S3). Avenida Erasmo Braga, 118 – 6o andar – Castelo – Rio de Janeiro – CEP 20 020-000. Tel (+55) 21 2333-2000. Fax (+55) 21 2332-6611. e-mail: [central.atendimento@faperj.br](mailto:central.atendimento@faperj.br). Website <http://www.faperj.br>. The funders had no role in study design, data collection and analysis, decision to publish, or preparation of the manuscript.

**Trial status**

Not yet recruiting. Start date: January 2022. Expected completion date: December 2024.

**Supporting information captions**

S1. Ethics committee approval letter.

S2. SPIRIT checklist.

S3. Proof of external funding.

S4. Study protocol approved by the ethics committee in the original language.

S5. Study protocol approved by the ethics committee in English.
